# Insomnia severity and daytime sleepiness in caregivers of advanced age

**DOI:** 10.3389/frsle.2024.1404684

**Published:** 2024-07-11

**Authors:** Lucy A. Webster, Talha Ali, Jody Sharninghausen, Alexandra M. Hajduk, Thomas M. Gill, Brienne Miner

**Affiliations:** ^1^Division of Psychiatry, University College London (UCL), London, United Kingdom; ^2^Centre for Health Services Studies, University of Kent, Canterbury, United Kingdom; ^3^Department of Internal Medicine, Yale University School of Medicine, New Haven, CT, United States

**Keywords:** aging, caregiving, insomnia, daytime sleepiness, advanced age

## Abstract

**Objective:**

Aging-related changes and caregiver burden may increase the risk for sleep disturbances among older caregivers, yet few studies have examined the prevalence of insomnia and daytime sleepiness in this group. We examined the relationship of caregiver status with insomnia and daytime sleepiness among persons of advanced age (>75 years of age).

**Design:**

Cross-sectional.

**Setting:**

Community.

**Participants:**

Yale Precipitating Events Project participants (n=383, mean age 84.4 years, 67% female, 12% African American, 25% caregivers).

**Measurements:**

Caregivers were persons who reported caring for another person in the past week or caring for/helping a friend or relative >4 times in the past month. We examined the correlates of caregiver status, including demographic (age, sex, race/ethnicity, education, marital status), psychosocial (living alone, Medicaid eligibility, depressive symptoms, social support, hours volunteered), and physical (obesity, chronic conditions, medication use, self-rated health status, physical activity, functional disability, cognitive impairment) factors. We used the Insomnia Severity Index (ISI) to establish insomnia severity (ISI score 0–28) or clinically significant insomnia symptoms (ISI ≥8). We used the Epworth Sleepiness Scale to establish daytime sleepiness (ESS score 0–24) or hypersomnia (ESS ≥10). In nonparametric multivariable regression analyses, we examined the relationship of caregiver status with insomnia or daytime sleepiness.

**Results:**

Compared to non-caregivers, caregivers were younger, more educated, less likely to be Medicaid eligible and had lower rates of depression, obesity, poor self-rated health, low physical activity, functional disability, and cognitive impairment. Mean ISI and ESS scores were in the normal range and similar among non-caregivers and caregivers (ISI scores of 6.9±5.6 and 6.9±5.4, and ESS scores of 6.4±4.7 and 6.1±4.3, in non-caregivers and caregivers, respectively). Multivariable-adjusted models demonstrated no significant differences in ISI and ESS scores between non-caregivers and caregivers (regression coefficients for ISI and ESS scores of −0.01 [95% CI = −1.58, 1.21] and −0.10 [−1.05, 1.21], respectively).

**Conclusion:**

When compared to older non-caregivers, older caregivers had more advantageous demographic, psychosocial, and physical profiles and had similar levels of insomnia and daytime sleepiness on standardized questionnaires. Future research is needed to elucidate contextual factors (e.g., caregiving intensity and care partner disease) that may increase the risk of sleep disturbances among caregivers of advanced age.

## Introduction

One in five caregivers in the United States is 65 years or older (Caregiving in the U. S., 2024). Due to the aging of our society, a larger proportion of caregivers will be of advanced age (75 years or older) in the future (Redfoot et al., 2013; National Alliance for Caregiving., 2015). Older persons are more likely to suffer from symptoms of insomnia and daytime sleepiness (Miner and Kryger, 2017; Miner et al., 2018, 2019), with prevalence rates of insomnia and daytime sleepiness as high as 50% and 46%, respectively among persons aged 75 to 84 years of age (National Sleep Foundation Sleep in America Poll, 2019. While referenced in 2019, the Sleep in America Poll was conducted in 2003). Caregiving may further increase this risk (Rowe et al., 2008; Koyanagi et al., 2018; Maun et al., 2019), but sleep complaints are frequently unrecognized and untreated in caregivers (Byun et al., 2016). Insomnia and daytime sleepiness are associated with adverse cognitive and functional outcomes (Foley et al., 1995, 2004; Ohayon, 2002; Ohayon and Vecchierini, 2002; Gooneratne et al., 2003; Ozminkowski et al., 2007; Vaz Fragoso and Gill, 2007; Kaufmann et al., 2013), and may impair older adults in their role as caregivers to the detriment of the care recipient. As caregivers provide immense social and economic benefits to our society (Colombo et al., 2011), it is imperative that we understand whether sleep disturbances represent a modifiable target to support them in their caregiving role.

Evidence on the relationship between sleep and caregiving among younger and middle-aged caregivers suggests that three quarters of caregivers report poor sleep quality (Byun et al., 2016). Evidence of sleep-disruptive symptoms among older caregivers, however, has been mixed. While certain studies suggest that caregivers are more likely to report sleep problems, especially if they provide a higher intensity of care (Rowe et al., 2008; Koyanagi et al., 2018; Maun et al., 2019), other studies have found that older caregivers do not always have more sleep problems than those not providing care (Kochar et al., 2007; Fredman et al., 2013; Song et al., 2017). Across these studies, the main measure used was the Pittsburgh Sleep Quality Index, which, while assessing insomnia-related symptoms, is a global measure of sleep quality (Buysse et al., 1989). One study in women veterans with a mean age of 52 years examined scores on the Insomnia Severity Index (ISI) (Song et al., 2021), a validated measure of insomnia that assesses a clinically significant threshold for insomnia symptoms as well as overall insomnia severity (Bastien et al., 2001; Morin et al., 2011). To our knowledge, no studies have examined scores on the ISI among caregivers of advanced age. A previous study investigated daytime sleepiness in older caregivers, who had worse daytime sleepiness on the Epworth Sleepiness Scale (ESS). However, the sample was relatively young, with an average age of 70.7 years (Rowe et al., 2008).

Using data from the Precipitating Events Project (PEP) (Gill, 2014) we examined the association of caregiver status among persons of advanced age (mean 84.4 years) with symptoms of insomnia and daytime sleepiness. Because aging is associated with an increasing prevalence of sleep disturbances (Miner and Kryger, 2017), and caregiving may serve as an additional precipitant for sleep-wake symptoms (Rowe et al., 2008; Koyanagi et al., 2018; Maun et al., 2019), we hypothesized that older caregivers would have more insomnia and daytime sleepiness than older non-caregivers.

## Methods

This is a secondary analysis of existing data from participants in the Yale Precipitating Events Project (PEP), a longitudinal study of older adults in the Greater New Haven area of Connecticut (Gill, 2014). PEP enrolled adults who, at study entry, were 70 years or older and non-disabled in activities of daily living. The assembly of this cohort has been previously described (Gill, 2014). Participants were excluded if they had significant cognitive impairment with no available proxy or inability to speak English. The cohort of 754 participants was assembled in 1998–99. Comprehensive assessments were done every 18 months by trained research staff. Collection of sleep-related questionnaires began at the 90-month assessment (2005–07). The study was approved by the Yale University Human Investigation Committee, and all participants gave informed consent.

For the current analysis, we excluded any participants who were residing in a skilled nursing facility or requiring a proxy to provide information at the time of the 90-month assessment. Of the 754 original participants, 287 died and 25 withdrew before 90 months. Another 59 participants were excluded (53 were living in a skilled nursing facility, 3 required a proxy, and 3 did not complete the caregiver questions). Hence, the analytical sample for the current study included 383 participants (50.8% of the initial cohort).

### Demographic, psychosocial, and physical characteristics

Demographic factors included age, sex, race/ethnicity (non-Hispanic White race vs. other), education level (completed high school vs. not) and marital status (married vs. other [separated, divorced, widowed, never married]). Psychosocial factors included living status (alone vs. with others), Medicaid eligibility status, depressive symptoms (Center for Epidemiologic Studies Depression Scale score ≥16) (Kohout et al., 1993), social support (Medical Outcomes Study Social support questionnaire score 0–35; higher scores indicate more social support) (Sherbourne and Stewart, 1991), and hours worked/volunteered in the past week (item on the Physical Activity Scale for the Elderly [PASE]) (Washburn et al., 1993). Physical characteristics included obesity (BMI ≥30), number of chronic conditions (count of self-reported, physician-diagnosed medical conditions, including hypertension, myocardial infarction, heart failure, stroke, cancer [excluding minor skin cancer], diabetes, hip fracture, arthritis, and/or chronic lung disease), number of medications (prescribed and over-the -counter), self-rated health (rated as excellent or very good vs. good, fair, or poor), low physical activity (PASE score of < 64 for men or 52 for women) (Vaz Fragoso et al., 2009), functional disability (number of impairments in up to 12 basic, instrumental and mobility activities) (Gill, 2014), and cognitive impairment (Mini Mental State Examination score < 24) (Folstein et al., 1975).

### Measures of insomnia and daytime sleepiness

The Insomnia Severity Index (ISI) measures seven self-reported symptoms and consequences of insomnia, mapping onto the criteria for Insomnia disorder from the Diagnostic and Statistical Manual of Mental Disorders, Fourth Edition (Bastien et al., 2001; Morin et al., 2011). Scores on the ISI range from 0 to 28, with higher scores indicating increased insomnia severity (Bastien et al., 2001). Scores of 8 or higher have been used as a threshold for clinically significant insomnia symptoms, including among older populations (Bastien et al., 2001), with a sensitivity of 95.8% and 99.4%, and specificity of 78.3% and 91.8% for establishing insomnia cases in community and clinical samples, respectively (Morin et al., 2011).

The Epworth Sleepiness Scale (ESS) measures daytime sleepiness through eight items where participants report how likely they are to fall asleep in a variety of circumstances. Scores on the ESS range from 0 to 24, with higher scores indicating more severe daytime sleepiness (Johns, 1991, 1994). Scores of 10 or higher are used as a threshold to indicate hypersomnia, including among older populations (Goldstein et al., 2004; Carvalho et al., 2017).

### Measures of caregiving

There were two items on caregiving collected within the PEP data. Firstly, on the PASE (Washburn et al., 1993) there was one item which asked the question “*In the past week did you care for another person?”* and could be answered yes or no. Secondly, on the Social Activity Scale (adapted from the EPESE interview) (Huntley et al., 1993) there was an item which asked the question “*In the past month how many times did you care for/help a friend or relative?”* with four response options: not at all, < 1, 1–4, or >4 times in the past month. Caregivers in the current analysis were defined as those who answered yes to the PASE caregiving question or who reported providing care at least four times in the last month on the Social Activity Scale caregiving question.

### Statistical analysis

We compared demographic, psychosocial, and physical factors between non-caregivers and caregivers, using student *t*-test for continuous variables and chi-square test for categorical variables. Because scores for the ISS and ESS were not normally distributed, we used non-parametric regression analyses to compare non-caregivers and caregivers. We conducted unadjusted and adjusted non-parametric linear regressions for continuous outcomes (ISS and ESS scores) and logistic regressions for binary outcomes (ISI ≥8; ESS ≥10). In multivariable analyses, we adjusted for all factors that were significantly different at *p* ≤ 0.05 between non-caregivers and caregivers. Multivariable models were also extended to include an interaction term of depressive symptoms by caregiving status, given evidence from prior literature that the relationship between caregiving status and the outcomes of insomnia severity and daytime sleepiness may differ according to the presence of depressive symptoms in the caregiver (Kochar et al., 2007). All analyses were conducted using STATA version 17.

## Results

**Figure 1:**
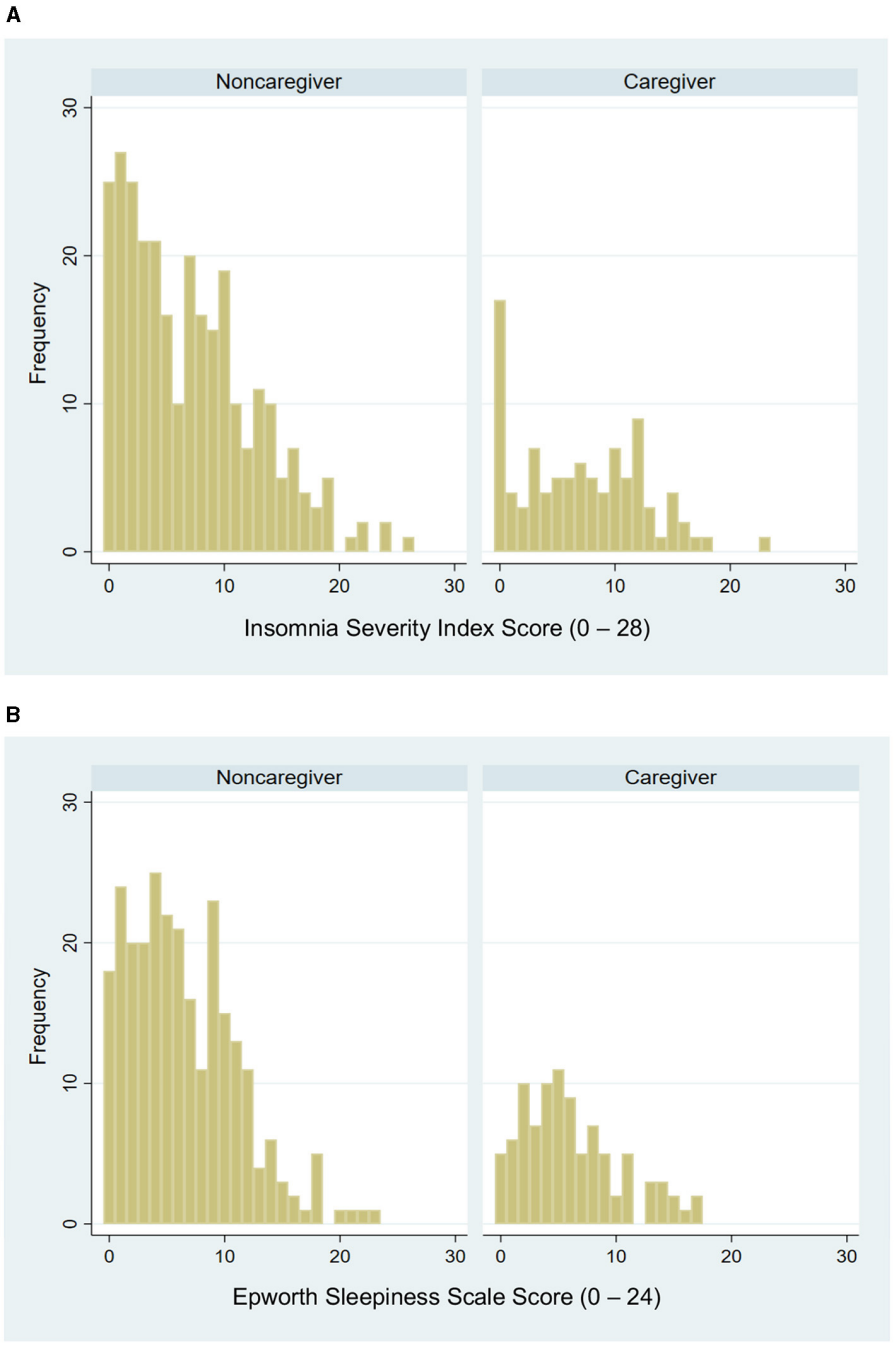
Insomnia Severity Index and Epworth Sleepiness Scale scores according to caregiving status. These figures show the distribution of scores among non-caregivers and caregivers, respectively, for the Insomnia Severity Index **(A)** and the Epworth Sleepiness Scale **(B)**.

Among the 383 participants, the average age was 84.4 years, 67% were female, and 88.3% identified as non-Hispanic White race (see Table 1). When comparing non-caregivers and caregivers, significant differences were seen in demographic, psychosocial, and physical factors. Caregivers (n=95) were younger, more likely to be male, more educated, more likely to be married, and less likely to live alone; less likely to be Medicaid eligible or to have depressive symptoms, and more likely to work or volunteer; and less likely to have low physical activity, functional disability or cognitive impairment. There were no significant differences in the ISI score or in the percentage of participants with an ISI ≥8 among non-caregivers and caregivers. Similarly, there were no significant differences in the ESS score or in the percentage of participants with an ESS ≥10 among non-caregivers and caregivers. The distribution of ISI and ESS according to caregiver status is shown in Figure 1.

**Table 1:** Clinical characteristics for the entire cohort and according to caregiver status.

**Characteristic**	**ALL (*N* = 383)**	**Caregiving status** ^**a**^		***p* value^b^**
		**Non-caregiver (*****n*** = **288; 75.1%)**	**Caregiver (*****n*** = **95; 24.9%)**	
		**Mean** ±**SD or n/N (%)**		
Age	84.4 ±4.6	84.9 ±4.7	82.9 ±3.9	< 0.001
Age range	78–102	78–102	78–97	-
Female sex	257 (67.1%)	201 (69.8%)	56 (58.9%)	0.05
Non-Hispanic White race	338 (88.3%)	255 (88.7%)	83 (87.4%)	0.76
Did not complete high school	115 (30.0%)	98 (34.4%)	17 (17.9%)	0.003
Married	147 (38.0%)	96 (33.0%)	51 (53.7%)	< 0.001
Living alone	176 (46.0%)	142 (49.1%)	34 (35.8%)	0.02
Medicaid eligible	40 (10.4%)	36 (12.7%)	4 (0.04%)	0.02
Depressive symptoms^c^	11.5 ±9.3	12.1 ±9.5	9.9 ±8.8	0.04
Social support score^d^	22.2 ±5.3	22.2 ± 5.5	22.1 ±4.9	0.97
Hours worked/volunteered in past week^e^	1.6 ±5.1	1.2 ±4.0	2.8 ±7.4	0.01
Obese^f^	73 (19.1%)	60 (21.0%)	13 (13.7%)	0.12
Number of chronic conditions^g^	2.3 ±1.2	2.4 ±1.3	2.1 ±1.1	0.08
Number of medications^h^	9.1 ±3.8	9.2 ±3.6	9.1 ±4.2	0.73
Self-rated health is excellent or very good	112 (29.2%)	77 (26.5%)	35 (36.8%)	0.06
Low physical activity^e^	208 (54.3%)	188 (65.6%)	20 (21.1%)	< 0.001
Functional disability^j^	3.5 ±3.2	4.1 ±3.4	1.9 ±1.9	< 0.001
Cognitive impairment^k^	64 (16.7%)	58 (20.6%)	6 (6.3%)	0.002
ISI score	6.9 ±5.5	6.9 ±5.6	6.9 ±5.4	0.99
ISI ≥8	161 (42.0%)	118 (40.5%)	43 (45.3%)	0.49
ESS score	6.3 ±4.6	6.4 ±4.7	6.1 ±4.3	0.56
ESS ≥10	82 (21.4%)	64 (22.0%)	18 (18.9%)	0.33

ESS, Epworth Sleepiness Scale; ISI, Insomnia Severity Index.

^a^Answered “yes” to Physical Activity Scale for the Elderly [PASE] question 25 (“In past week did you care for another person?”) OR “more than 4 times” to the Social Activity Scale [SAS] question (“In past month how many times did you care for/help a friend or relative?”).

^b^Compared between caregivers and non-caregivers, using Student t-test or Chi square-test, as appropriate.

^c^Center for Epidemiologic Studies Depression scale score ≥ 16.

^d^Medical Outcomes Study Social Support questionnaire score (0–35).

^e^From PASE question 9b.

^f^BMI ≥ 30.

^g^Count of self-reported, physician-diagnosed medical conditions (including hypertension, myocardial infarction, heart failure, stroke, cancer [excluding minor skin cancer], diabetes, hip fracture, arthritis, and/or chronic lung disease).

^h^Includes prescribed and over-the-counter medications.

^i^PASE score of < 64 (men) or < 52 (women).

^j^Number of impairments in up to 12 basic, instrumental, and mobility activities.

^k^Mini Mental State Examination score < 24.

In unadjusted and multivariable models adjusting for factors that differed significantly between non-caregivers and caregivers (i.e., age, sex, education, marital status, living alone, Medicaid eligibility, depressive symptoms, hours worked or volunteered, low physical activity, functional disability, cognitive impairment), caregiving status was not associated with insomnia severity (adjusted regression coefficient = −0.01 [95% CI = −1.58, 1.21]) or daytime sleepiness (adjusted regression coefficient = −0.10 [−1.05, 1.21]) (see Table 2). Similarly, caregivers did not have higher odds of insomnia or hypersomnia than non–caregivers in unadjusted or adjusted logistic regression models (adjusted ORs of 1.20 [0.65, 2.25] and 1.11 [0.54, 2.30] for insomnia and hypersomnia, respectively; see Table 3). There was no evidence that depressive symptoms in the caregiver were associated with significant differences in insomnia (OR = 1.05 [0.36, 3.11]; *n* = 377) or hypersomnia (OR = 0.98 [0.32, 3.04]; *n* = 357).

**Table 2:** Relative risk of insomnia severity and daytime sleepiness among caregivers^*^.

**Outcome**	**Unadjusted regression coefficient (95% CI)^a^**	**Adjusted regression coefficient (95% CI)^b^**
Insomnia severity (ISI 0-28)	−0.001 (−0.17, 0.20)	−0.01 (−1.58, 1.21)
Daytime sleepiness (ESS 0-24)	−0.05 (−0.23, 0.14)	−0.10 (−1.05, 1.21)

CI, confidence interval; ESS, Epworth Sleepiness Scale; ISI, Insomnia Severity Index.

^*^Answered “yes” to Physical Activity Scale for the Elderly [PASE] question 25 (“In past week did you care for another person?”) OR “more than 4 times” to the Social Activity Scale [SAS] question (“In past month how many times did you care for/help a friend or relative?”).

^a^From non-parametric regression models. Reference group is non-caregivers.

^b^From multivariable models adjusted for age, sex, education, marital status, living alone, Medicaid eligibility, depressive symptoms, number of hours worked/volunteered, low physical activity, functional disability, and cognitive impairment.

**Table 3:** Odds of insomnia and hypersomnia among caregivers^*^.

**Outcome**	**Unadjusted Odd Ratio (95% CI)^a^**	**Adjusted Odds Ratio (95% CI)^b^**
Insomnia (ISI ≥ 8)	1.18 (0.74, 1.89)	1.20 (0.65, 2.25)
Hypersomnia (ESS ≥ 10)	0.75 (0.42, 1.35)	1.11 (0.54, 2.30)

CI, confidence interval; ESS, Epworth Sleepiness Scale; ISI, Insomnia Severity Index.

^*^Answered “yes” to Physical Activity Scale for the Elderly [PASE] question 25 (“In past week did you care for another person?”) OR “more than 4 times” to the Social Activity Scale [SAS] question (“In past month how many times did you care for/help a friend or relative?”).

^a^From logistic regression models. Reference group is non-caregivers.

^b^From multivariable models adjusted for age, sex, education, marital status, living alone, Medicaid eligibility, depressive symptoms, number of hours worked/volunteered, low physical activity, functional disability and cognitive impairment.

## Discussion

Our study is one of the first to examine symptoms of insomnia and daytime sleepiness in caregivers of advanced age and to include a validated measure of insomnia symptoms. We found significant demographic differences and more advantageous psychosocial and physical health profiles in caregivers as compared to non-caregivers. Despite advanced age and substantial medical comorbidity in this cohort, scores on the ISI and ESS were in the normal range and did not differ by caregiver status.

Our findings differ from several previously published studies of sleep in caregivers, which demonstrated worse sleep and higher levels of daytime sleepiness in caregivers as compared to non-caregivers (Rowe et al., 2008; Koyanagi et al., 2018; Maun et al., 2019). These differences are likely due to variations in the population studied and study design. A systematic review of sleep in middle-aged caregivers found that caregiver health status and symptoms, including depression and anxiety, were associated with worse sleep in the caregiver (Byun et al., 2016). The caregivers in our study, on the other hand, reported better health status and lower levels of depression than non-caregivers. Previous studies among older caregivers focused on specific, high-intensity situations. For example, a prior study found worse sleep in older caregivers who co-resided with their care partner, with caregiving over 50 h per week and continuous co-resident caregiving increasing the odds of sleep problems (Maun et al., 2019). Worse sleep in older adult caregivers of family members with dementia, especially those with night-time activity, has also been well-described (Hope et al., 1998; Rowe et al., 2008). In contrast, we used a broad definition for caregiving in our study, which included having helped or cared for a friend or relative at least once a week. There were no criteria for hours per week, intensity of caregiving duties performed, co-habitation, or disease of the care partner, all of which could significantly shape the sleep opportunity and experience of the caregiver.

In addition to caregiving intensity and care partner disease, other important conditions affecting sleep in the caregiver are caregiver stress level and depression. A study of older women caregivers (average age 83 years) found that, while caregiving status alone was not associated with sleep problems, among high-intensity caregivers, those with high stress levels had significantly longer wake after sleep onset, and high stress was also significantly associated with worse sleep regardless of caregiving status (Song et al., 2017). Similarly, among middle-aged women veterans, there was no difference in ISI scores according to caregiver status, but caregivers were more likely than non-caregivers to report stress as a cause of poor sleep (Song et al., 2021). In our study, there was no measure of stress level. In contrast to previous work (Kochar et al., 2007), we did not find evidence of worse sleep among caregivers with higher levels of depression.

The advanced age of our cohort may have influenced our results in several ways. The average age of caregivers in our study was 84 years; by comparison, average age was in the 70s in studies showing worse sleep in older caregivers than non-caregivers (Rowe et al., 2008; Maun et al., 2019). A prior study of family dementia caregivers found that sleep difficulties were significantly related to role overload in adult children but not spousal caregivers (Liang et al., 2020). While we cannot confirm that most caregivers in our study were caring for a spouse, the older age and the fact that over half of caregivers were married makes this more likely. It is also possible that some caregivers in our study may have been caring for a child (e.g., grandchild). Among caregivers with poor sleep, caring for a child has been associated with less depression and anxiety than caring for an adult (Song et al., 2018). Research has shown younger age in the caregiver to be associated with self-reported depression and loneliness (Musich et al., 2017), which may relate to juggling family needs and career with caregiving and precipitate sleep problems. Conversely, caregivers of advanced age may have fewer competing social demands and relative protection from role overload as a mechanism for sleep disturbance. Furthermore, older informal caregivers report less psychological distress compared to younger informal caregivers (Kabia et al., 2022). Another consideration is that the ISI and ESS, which were validated in younger age groups (Johns, 1991, 1994; Bastien et al., 2001; Morin et al., 2011), may not perform well among persons of advanced age. The ISI was validated in two groups (younger and older), but the mean age of the older group was 65 years (range 55–84) (Bastien and Bonnet, 2001), while the ESS was validated among persons with ages ranging 20s−60s (Johns, 1991). In the ISI, questions five through seven in particular, which ask participants to report whether sleep problems are noticeable, worrying, or interfering with daily function, may not be relevant for persons with fewer daytime demands and a more flexible schedule. To investigate this, we did a sensitivity analysis examining average ISI scores among caregivers vs. non-caregivers after omitting questions five through seven. However, we found no significant difference in scores between caregivers and non-caregivers on the abbreviated ISI (i.e., caregiver score was 0.04 [-0.09, 0.15] points higher; *p* = 0.578). Finally, it is possible that there is an age-related decrease in the report of insomnia and daytime sleepiness (Miner et al., 2018, 2019). Prior work suggests age is associated with lower ESS scores (Li et al., 2018; Berger et al., 2021), and that older persons report milder levels of insomnia and daytime sleepiness despite having more severe sleep disorders (Vaz Fragoso et al., 2015; Iannella et al., 2019).

Gender differences and healthy survivor effects may also explain our results. Caregivers in our sample were more likely to be male, who tend to report lower rates of insomnia than women (Li et al., 2018). In addition, previous studies have found that male caregivers tended to have better physical health and experience less burden when compared to older female caregivers (Savela et al., 2022; Spatuzzi et al., 2022). Finally, older caregivers of all genders are more likely to be selected for the caregiving role because they are healthier (Mikkola et al., 2021).

Differences in method of assessment of sleep-related symptoms may have contributed to differences in findings for the current study. We used the ISI, which focuses on the cardinal symptoms of insomnia (i.e., difficulty with sleep initiation, maintenance, and/or early morning awakenings) as well as its consequences, mapping onto the DSM-IV criteria for Insomnia disorder (Bastien et al., 2001; Morin et al., 2011). The ISI has been used to measure insomnia in dementia caregivers (Jiménez-Gonzalo et al., 2021), in caregivers of people receiving hospice care (Starr et al., 2022), and in middle-aged women veterans who were caregivers (Song et al., 2018), but not previously, to our knowledge, among caregivers of advanced age. Most existing studies examining sleep in caregivers have used measures of sleep quality, which describes an individual's self-satisfaction with all aspects of the sleep experience (Nelson et al., 2022). However, several studies have assessed insomnia symptoms, albeit without a validated questionnaire, in caregivers vs. non-cargivers (Kochar et al., 2007; Koyanagi et al., 2018; Maun et al., 2019). Thus, it seems that factors related to caregiver characteristics and caregiving intensity are more likely than the method of sleep assessment to explain the difference in the current study as compared to previous findings. The only other study examining daytime sleepiness via the Epworth Sleepiness Scale among older caregivers supports this theory. They found higher levels of daytime sleepiness in caregivers than non-caregivers, but the population studied was younger (average age of 71 years) and they specifically selected caregivers who provided direct care to a person with dementia with night-time activity (Rowe et al., 2008).

There are a growing number of older caregivers worldwide due to demographic shifts and the aging of society (Broese van Groenou and De Boer, 2016; Hillman et al., 2018). Caregiving in older age may add meaning in life and help caregivers to maintain an active lifestyle (Suntai, 2021). Research has shown that loneliness and social isolation in community-dwelling older adults are linked with sleep problems (McLay et al., 2021; Qi et al., 2023), so the act of caregiving may actually provide sleep protection via increased social connectivity and sense of purpose. While sleep issues increase with older age, a study of older adults (average age over 75) found that volunteer participation mitigated the relationship between insomnia and poor subjective wellbeing (Wang et al., 2022), further supporting potential health benefits of informal caregiving in this group. Our results indicate the need to consider not only burdens, but also benefits of the caregiving role.

Strengths of our study include use of validated measures of insomnia and daytime sleepiness in caregivers of advanced age. The PEP dataset itself is also rich with many outcomes, allowing us to account for important sociodemographic and clinical variables. Limitations include the broad definition of caregiving used in our study and lack of information on caregiving intensity and context.

In conclusion, our findings suggest that among persons with advanced age the caregiver role itself is not associated with higher levels of insomnia or daytime sleepiness. Caregivers of advanced age in our study had more advantageous sociodemographic, psychosocial and physical health profiles than non-caregivers. Notably, these older caregivers may have relative protection from sleep ramifications of caregiving and draw social benefits from caregiving. Caregivers of advanced age represent a vital but understudied workforce in society. Future studies should explore the association of caregiving with certain characteristics of the older caregiver (e.g., stress level) and care context (e.g., intensity of caregiving duties) when evaluating sleep disturbances in caregivers, and potential benefits of the caregiver role should also be considered.

## Data availability statement

The datasets presented in this article are not readily available because participants interested in using the study data should contact the principal investigator for the study, TG. Requests to access the datasets should be directed to thomas.gill@yale.edu.

## Ethics statement

The studies involving humans were approved by Yale University Human Investigation Committee. The studies were conducted in accordance with the local legislation and institutional requirements. The participants provided their written informed consent to participate in this study.

## Author contributions

LW: Conceptualization, Data curation, Formal analysis, Investigation, Methodology, Project administration, Software, Validation, Visualization, Writing – original draft, Writing – review & editing. TA: Conceptualization, Investigation, Methodology, Writing – original draft, Writing – review & editing. JS: Writing – original draft, Writing – review & editing. AH: Writing – original draft, Writing – review & editing, Conceptualization, Data curation, Investigation, Methodology, Supervision. TG: Conceptualization, Data curation, Investigation, Methodology, Supervision, Writing – original draft, Writing – review & editing, Funding acquisition, Resources. BM: Conceptualization, Data curation, Funding acquisition, Investigation, Methodology, Resources, Supervision, Writing – original draft, Writing – review & editing, Formal analysis, Project administration, Software, Validation, Visualization.
